# Editorial on the Special Issue “Modification of Hydrogels and Their Applications in Biomedical Engineering”

**DOI:** 10.3390/gels9040263

**Published:** 2023-03-23

**Authors:** Yanen Wang, Qinghua Wei

**Affiliations:** 1 Department of Indurstry and Engineering, School of Mechanical Engineering, Northwestern Polytechnical University, Xi’an 710072, China; 2 Bio-Additive Manufacturing University-Enterprise Joint Research Center of Shaanxi Province, Northwestern Polytechnical University, Xi’an 710072, China

Hydrogels and hydrophilic polymer networks play an important role in biomedical engineering due to their good biocompatibility, biodegradability, hydrophilicity, and mechanical properties, similarly to some soft tissues. However, single-component hydrogels usually do not meet the basic functional requirements of biomedical engineering, and modification is an effective way to improve the properties of hydrogels. Currently, a variety of multifunctional hydrogels with excellent performance have been developed and greatly promote the application of hydrogels in biomedical engineering. This Special Issue focuses on the modification of hydrogels and their applications in biomedical engineering, aiming to provide a reference for scholars in related fields. Up until now, ten research articles and one review article have been collected, which mainly involve the application of hydrogels in biomedical fields such as phantoms [1], wound healing [2,3], skin [4], cartilage [5,6], muscle [7], and drug loading [8,9,10].

The use of phantoms offers a promising solution for the simulation of biological bodies. As tissue phantoms, mimicking the mechanical properties of soft living tissues is the basic requirement. In this regard, Tejo-Otero and his coauthors [1] tested the mechanical properties of a wide range of organs (e.g., liver, heart, kidney, as well as brain), hydrogels (e.g., agarose, polyvinyl alcohol (PVA), Phytagel (PHY), and methacrylate gelatin (GelMA)); the correlation between the mechanical properties of the organs and the different materials was obtained. This provides a reference for the selection of matrix materials for soft tissue models. In terms of wound healing, Chiangnoon et al. [2] used electron beam (EB) irradiation to develop antibacterial hydrogel sheet dressings from poly(vinyl alcohol) (PVA) and silver nanoparticles (AgNPs) in a two-step process and evaluated their bactericidal efficacy, as well as the AgNP’s release. This research provides a convenient platform for the preparation of AgNP-loaded hydrogel dressings that can be further developed for wound healing. Meanwhile, Afrin et al. [3] prepared a semi-IPN hydrogel by combining the natural polymer cellulose nanocrystal (CNC) and the synthetic polymer polyethylene glycol (PEG) and poly (N, N′-dimethyl acrylamide) (PDMAA). The semi-IPN hydrogel loaded with the antibiotic drug, gentamicin, also shows excellent wound-healing properties. In a work related to electronic skin, Chen et al. [4] successfully prepared composite hydrogels based on polyvinyl alcohol (PVA), gelatin (GEL), oxidized sodium alginate (OSA), graphene oxide (GO), and single-walled carbon nanotubes (SWNTs). The prepared composite hydrogel exhibits good flexibility, self-healing, and conductive performance, which shows great potential for applications in electronic skin. To obtain an ideal cartilage substitute, Jalageri et al. [5] blended PVA and PVP with a one-dimensional hydroxyapatite nanorod (HNr) and synthesized a PVA/PVP/HNr composite hydrogel via the freeze–thaw method. The experimental results confirm that nanohydroxyapatite-reinforced PVA/PVP hydrogels are promising alternatives for next-generation cartilage substitutes. In another related work, Manferdini et al. [6] analyzed two different concentrations (1:1 and 1:2) of VitroGel^®^ (VG) hydrogels without (VG-3D) and with arginine–glycine–aspartic acid (RGD) motifs (VG-RGD) and verified their ability to support the chondrogenic differentiation of encapsulated human adipose mesenchymal stromal cells (hASCs). They found that both hydrogels, at different concentrations, and the presence of RGD motifs significantly contributed to the chondrogenic commitment of laden hASCs. Regarding the application of hydrogels in muscle tissue, Mubarok et al. [7] evaluated the influence of physicochemical property dynamics in the gelatin that possess phenol groups (Gelatin-Ph) and hydrogels to regulate the myogenesis in vitro, and the myogenesis was successfully tuned by changes in the physicochemical properties of Gelatin-Ph hydrogel mediated by H_2_O_2_. This provides a reference for the regeneration and repair of muscle tissue. Hydrogels also have important applications in the controlled release of drugs. Chittasupho et al. [8] developed shape memory gels containing phytosomes as a delivery system for fresh (VFL) and dry (VDL) leaf extracts of *Nicotiana tabacum* var. Virginia. The results showed that the VDL and VFL phytosomes dispersed in shape memory gels could be considered as a promising therapeutic delivery system to protect the skin from oxidation and reactive oxygen species. Kim and his co-authors [9] prepared a hyaluronic acid (HA)–collagen hybrid hydrogel to investigate the effect of fibroblast growth factor (FGF)-2 and platelet-derived growth factor (PDGF)-BB on human pulp regeneration. The results show that the optimal concentration of FGF-2 and PDGF-BB for pulp cell proliferation was 100 ng/mL and that the HA–collagen hybrid hydrogel has the potential as a controlled release delivery system for FGF-2 and PDGF-BB. Srikhao et al. [10] fabricated hydrogel beads for use as a drug delivery system based on basil seed mucilage (BSM), sodium alginate (SA), and magnetic particles (MPs). Based on the results of this work, BSM/SA/MPs hydrogel beads were considered to have the potential to be used as a pH-sensitive alternative material for drug delivery in colon-specific systems. In addition, smart hydrogels based on poly(N-isopropyl acrylamide) (PNIPAM) have been applied in various biomedical applications such as drug delivery, tissue engineering, and wound dressings due to their distinct thermoresponsive features, such as being close to a lower critical solution temperature (LCST). Nevertheless, they have intrinsic shortcomings, such as poor mechanical properties, limited drug loading capacity, and poor biodegradability. Ansari et al. [11] reviewed the latest developments in functional PNIPAM-based smart hydrogels for various biomedical applications and summarized the challenges and opportunities in this emerging field of research.

This Special Issue offers a real insight into the progress made in the application of hydrogel in biomedical engineering. There is every reason to believe that with the efforts of researchers, multifunctional hydrogels will play an increasingly important role in biomedical engineering in the future.
